# Investigation of biphenyl enamines for applications as p-type semiconductors

**DOI:** 10.1098/rsos.230260

**Published:** 2023-07-26

**Authors:** Matas Steponaitis, Vygintas Jankauskas, Egidijus Kamarauskas, Vida Malinauskienė, Smagul Karazhanov, Tadas Malinauskas, Vytautas Getautis

**Affiliations:** ^1^ Department of Organic Chemistry, Kaunas University of Technology, Radvilenu pl. 19, 50254 Kaunas, Lithuania; ^2^ Institute of Chemical Physics, Vilnius University, Sauletekio av. 9, 10222 Vilnius, Lithuania; ^3^ Institute for Energy Technology (IFE), P.O Box 40, NO 2027, Kjeller, Norway

**Keywords:** one-step synthesis, biphenyl enamines, organic semiconductors, charge transport

## Abstract

Due to the ease of synthesis and the ability to easily tune properties, organic semiconductors are widely researched and used in many optoelectronic applications. Requirements such as thermal stability, appropriate energy levels and charge-carrier mobility have to be met in order to consider the suitability of an organic semiconductor for a specific application. Balancing of said properties is not a trivial task; often one characteristic is sacrificed to improve the other and therefore a search for well-balanced materials is necessary. Herein, seven new charge-transporting biphenyl-based enamine molecules are reported. The new materials were synthesized using a simple one-step reaction without the use of expensive transition metal catalysts. It was observed that subtle variations in the structure lead to notable changes in the properties. Materials exhibited high thermal stability and relatively high carrier drift mobility, reaching 2 × 10^−2^ cm^2^V^−1^ s^−1^ (for **BE3**) at strong electric fields. Based on the results, three materials show the potential to be applied in organic light emitting diodes and solar cells.

## 1. Introduction

Since the first device employing an organic semiconductor (OS) was constructed in the second half of the last century [1], many new applications for organic charge-transporting materials have emerged such as: organic light emitting diodes (OLED) [2], organic field effect transistors (OFET) [3], various solar cells (SCs) [4–6] etc. Devices are usually made of many different layers of OSs that perform different functions, such as charge transfer, light emission, light absorption etc. [6,7]. Different functions mean different requirements for OSs in terms of charge transport, thermal, film-forming properties, energy levels and emission characteristics. For example, OSs used in OLEDs as the emitter need to possess high internal quantum efficiency [8,9], while OSs performing charge-transporting functions in OLEDs, perovskite SCs (PSCs), antimony-based SCs or organic SCs (OSCs) need to have appropriate energy levels, relatively high charge-carrier mobility and the ability to form high-quality thin films [8–13]. Most of the recent research on OFETs is orientated towards sensing applications; therefore the main requirements for OSs in OFETs are: efficient charge transport and the ability to capture molecules of an analyte [3,14]. Similar properties to OSs used in OLEDs are expected from charge-transporting materials used in PSCs, antimony-based SCs and OSCs [15,16].

OSs are crucial in achieving the best possible performance of the above-mentioned devices; removing any of the charge-transporting layers usually leads to a significant loss of overall efficiency [17–19]. Besides the necessary properties, if the device is expected to be commercialized, the semiconductor must be relatively inexpensive [6,20]. The variety of ways organic molecules can be synthesized and modified gives flexibility to choose the most cost-efficient method to obtain OSs, not to mention the freedom to manipulate properties of the materials by making small changes to their molecular structure. Furthermore, most OSs can be solution processed and deposited on top of flexible substrates, lowering the fabrication cost and widening the scope of applications of various devices, e.g. lightweight and flexible SCs could be mounted on glass panels of high-rise buildings to power them fully or partially [21].

The need for organic charge-transporting materials with appropriate properties remains high, despite the constant efforts of the research community. In this work we present seven new enamine-based hole-transporting materials (HTMs) synthesized in a simple one-step reaction from commercially available materials without the use of expensive transition metal catalysts. It was observed that subtle variations in the structure of biphenyl precursors lead to notable changes in the properties of the new OSs. All HTMs exhibited high thermal stability and relatively high carrier drift mobility, reaching 2 × 10^−2^ cm^2^ V^−1^ s^−1^ (**BE3**) at strong electric fields, rivaling some of the best-known enamine-based HTMs [22–24].

## 2. Results and discussion

### 2.1. Synthesis

**Scheme 1.**
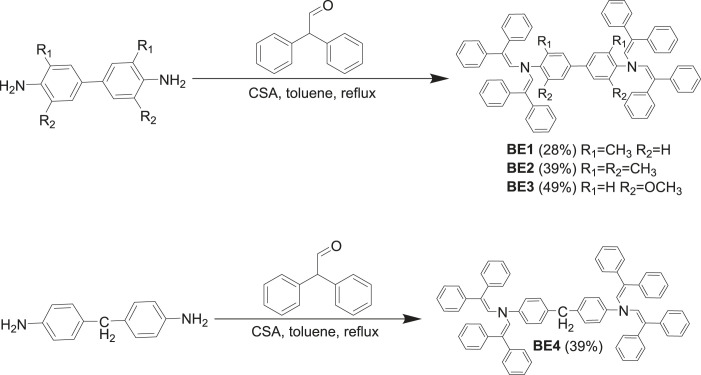
Synthesis of biphenyl enamines **BE1**–**BE4**.

Biphenyl derivatives **BE1**, **BE2**, **BE3** and their analogue **BE4** were synthesized via facile one-step camphorsulfonic acid (CSA) catalyzed diphenylacetaldehyde condensation reaction with the corresponding aromatic diamines (scheme 1) [25]. Although all four target compounds were isolated in similar yields, it still can be noticed that the presence of a stronger electron-donating group in the benzene ring increases reactivity of aromatic diamine and therefore a higher yield of **BE3** is obtained.

**Scheme 2.**
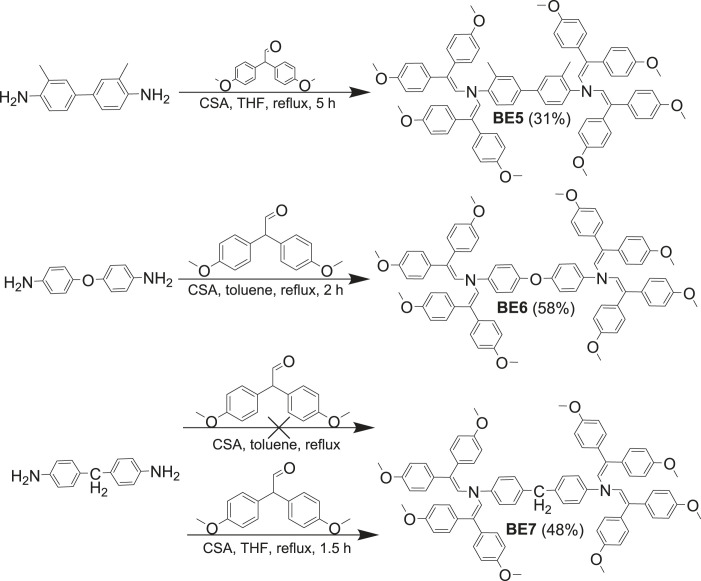
Synthesis of enamines **BE5**, **BE6** and **BE7**.

The condensation of *o*-tolidine and 2,2-bis(4-methoxyphenyl)acetaldehyde in toluene was unsuccessful; therefore, the solvent was changed to THF to give biphenyl-based enamine **BE5** (scheme 2). The reactions of biphenyl derivatives of *m*-tolidine, 3,3′,5,5′-tetramethylbenzidine and *o*-dianisidine with the aforementioned aldehyde containing methoxy groups did not yield the desired products (electronic supplementary material, scheme S1). An assumption can be made that the reactivity of 2,2-bis(4-methoxyphenyl)acetaldehyde is slightly lower than diphenylacetaldehyde due to strong electron-donating groups in the benzene ring. Nevertheless, this effect on carbonyl group reactivity should be mild as this group is not connected directly to aromatic system. Strangely enough, aldehyde condensation with 3,3′-dimethoxybiphenyl-4,4′-diamine, which is a stronger nucleophile, did not give the desired product, while reaction with 3,3′-dimethoxybiphenyl-4,4′-diamine gave **BE5** in a moderate yield. The other reasonable explanation might be that the course of reaction is influenced by spatial constraints that are restricting the formation of corresponding tetrasubstituted products, as one of the aforementioned reactions gave a trisubstituted compound but no tetrasubstituted enamine.

Synthesis of oxydianiline-based enamine **BE6** was conducted by reacting 2,2-bis(4-methoxyphenyl) acetaldehyde with 4,4′-oxydianiline in the presence of CSA in toluene (scheme 2).

Use of toluene as the solvent for the condensation of 4,4′-methylenedianiline with the above-mentioned methoxy aldehyde did not yield the appropriate enamine **BE7** as the desired product. Similarly to **BE5**, aniline derivative **BE7** was obtained by changing the solvent to THF (scheme 2). The different outcome of the reactions in toluene or THF can be linked to the difference in solubility of reaction components in the corresponding solvent.

### 2.2. Thermal properties

**Figure 1.**
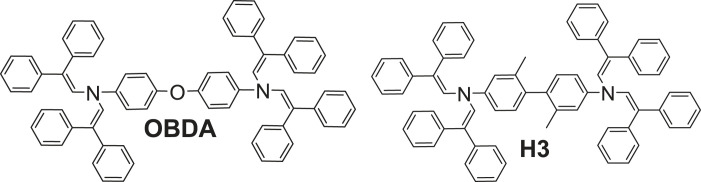
Chemical structures of OBDA and H3.

Thermal stability of materials is an important parameter to know as working conditions in devices vary and in some cases can reach up to 130°C [26]. Thermal stability of biphenyl enamines was measured using thermogravimetric analysis (TGA); the results are listed in table 1. For comparison, two known biphenyl derivatives 4,4′-oxybis[*N*,*N*-bis(2,2-diphenylethenyl)aniline] (OBDA) [25] and *N^4^*,*N^4^*,*N^4^*′,*N^4^*′-tetrakis(2,2-diphenylethenyl)–2,2′-dimethyl[1,1′-biphenyl]-4,4′-diamine (H3) (figure 1) [24] are also added to table 1.

**Table 1.** Thermal properties of compounds **BE1–BE7**, H3 and OBDA.

compound	*T*_g_*^a^*,°C	*T*_m_*^b^*,°C	*T*_cr_*^c^*,°C	*T*_dec_*^d^*,°C
H3	—	339	240	361
**BE1**	103	—	—	327
**BE2**	106	218	174	382
**BE3**	100	228	—	383
**BE4**	138	267	—	350
OBDA	139	295	244	405
**BE5**	63	198	159	215
**BE6**	131	289	—	361
**BE7**	130	—	—	366

^a^Determined by DSC: scan rate = 10°C min^−1^, N_2_ atmosphere; second run.

^b^Determined by DSC: scan rate = 10°C min^−1^, N_2_ atmosphere; first run.

^c^Determined by DSC: scan rate = 10°C min^−1^, N_2_ atmosphere.

^d^Onset of decomposition determined by TGA: heating rate = 10°C min^−1^, N_2_ atmosphere.

**Figure 2.**
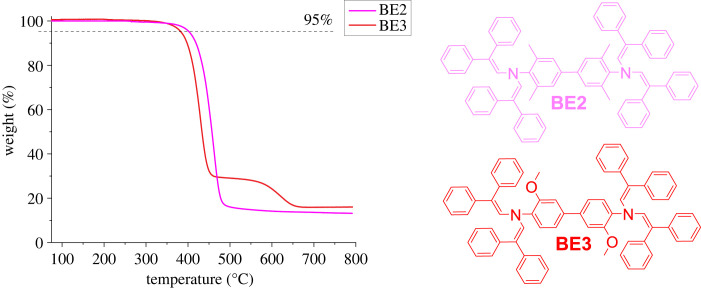
TGA curves of enamines **BE2** and **BE3**.

With the exception of **BE5**, all the tested HTMs showed a 5% weight loss at temperatures higher than 300°C, thereby confirming that they are sufficiently thermally stable for application in electronic devices. Furthermore, materials **BE2** and **BE3** demonstrated rapid weight loss, indicating rapid evaporation, which suggests they show potential for application in vacuum-deposited devices (figure 2).

The peculiar case of a relatively low stability of **BE5** could be explained by the spatial restraints due to the presence in the close proximity of large bis(4-methoxyphenyl)ethenyl substituents and the methyl groups of the biphenyl core that, when heated, might limit the movement of the separate parts of its molecule, thereby causing bond-breaking strains. The loss of more than 20% mass (electronic supplementary material, figure S1) coincides with the loss of a single bis(4-methoxyphenyl)ethenyl fragment.

**Figure 3.**
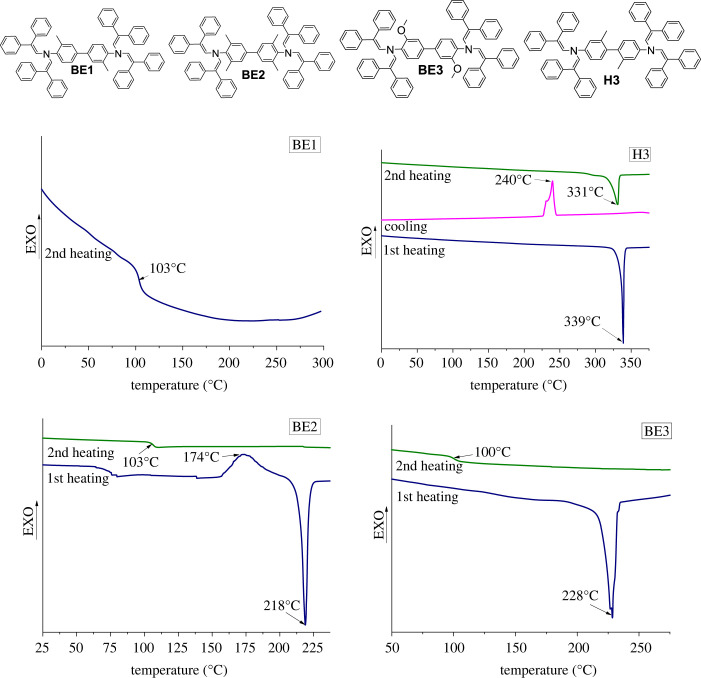
Structures of enamines **BE1**–**BE3** and H3 and their DSC curves.

To ensure the integrity of the device, it is important to choose materials that do not show phase transition within the operating temperatures of the device, e.g. OSs used in SCs are expected to withstand temperatures of at least 85°C without change in their performance [27]. For this purpose, differential scanning calorimetry (DSC) was used to determine melting (*T*_m_), glass transition (*T*_g_) and crystallization (*T*_cr_) temperatures. The DSC curves of biphenyl enamines **BE1**, **BE2**, **BE3** and H3 can be observed in figure 3. Enamine **BE1** is an amorphous compound with a *T*_g_ value of 103°C. Interestingly, due to the limited rotation around the bond connecting phenyl rings in the biphenyl fragment, a change of the position of methyl groups from *ortho* to *meta* has a significant effect on the thermal properties of compound H3, making it crystalline. The limitation is caused by methyl groups in the *meta* position together with diphenylethenyl substituents in H3, thereby making the molecule more rigid and hence leading to a greater tendency to crystallize.

DSC curves of **BE4** can be seen in electronic supplementary material, figure S2. The compound can exist in the crystalline state as well as in the amorphous state, as melting of the crystals can be seen during the first heating, and only *T*_g_ is observed during the second heating. In comparison, a known biphenyl derivative OBDA, where the methylene linker is replaced by oxygen, has a similar *T*_g_ of 140°C but roughly a 30°C higher *T*_m_ value (table 1).

DSC curves of compounds **BE5**–**BE7** can be observed in electronic supplementary material, figure S3. Biphenyl derivative **BE5**, as obtained after synthesis and purification, is partially crystalline, and a mixture of amorphous and crystalline material is observed; however, during the second heating, only *T*_g_ is seen (electronic supplementary material, figure S3a). Introduction of the oxygen linker in **BE6** results in material that can be crystalline, as observed during first heating, but also could form a stable amorphous state indicated by the occurrence of only glass transition during the second heating cycle (electronic supplementary material, figure S3b). Interestingly, due to the less rigid nature of the **BE7** molecule compared to **BE6,** changing the linker from oxygen to methylene leads to a compound that has only an amorphous state (electronic supplementary material, figure S3c).

### 2.3. Optical properties

**Figure 4.**
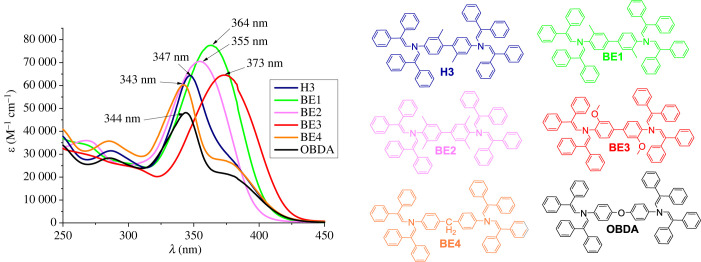
UV-Vis absorption spectra of **BE1**–**BE4**, H3 and OBDA in THF.

The light absorption spectra of biphenyl derivatives **BE1**–**BE4** were recorded in THF and are presented in figure 4; spectra of OBDA and H3 are shown for comparison. H3 absorption peaks at 347 nm, while the change of the position of the methyl groups from *meta* to *ortho* in **BE1** redshifts the absorption maximum by 17 nm. Changing the methyl groups in the *ortho* position of **BE1** to methoxy in **BE3** causes a slight batochromic shift due to the stronger donoric properties of the methoxy substituents. **BE2** with additional methyl substituents in the *ortho* position exhibits a hypsochromic shift compared to **BE1**, but to a smaller extent compared to H3. The shifts in the absorption maxima of biphenyl derivatives H3 and **BE2** could be explained by out-of-plane twisting of the phenyl rings of the biphenyl moiety when the methyl groups are in the *meta* position or stronger steric hindrances occurring when there are more than two substituents in the *ortho* position. This results in the reduction in size of the π-conjugated system, as the absorption maxima of H3 and **BE2** resemble those of **BE4** and OBDA that have their conjugation interrupted by the methylene group and oxygen atom, respectively.

The UV-Vis absorption spectra of methoxy-substituted aniline derivatives **BE5**–**BE7** are presented in electronic supplementary material, figure S4. The π-π* electron transition of compound **BE5** peaks at around 370 nm, while the other two materials display hypsochromic shifts of more than 20 nm. Similar to the case of OBDA and **BE4**, this could be explained by the interruption of conjugation by the oxygen or methylene linkers in **BE6** and **BE7**, respectively.

### 2.4. Photophysical and electrochemical properties

To evaluate the electrochemical properties of the synthesized compounds, cyclic voltammetry (CV) measurements in DCM were carried out. All the investigated materials demonstrated reversible oxidation; cyclic voltammograms of the tested HTMs are shown in electronic supplementary material, figure S6.

As mentioned earlier, one of the key parameters for choosing an OS for application in a device is the energy level of the material. Different architecture devices have their own requirements of energy levels for charge-transporting materials. In the case of OLEDs, antimony-based SCs, OSCs and triple cation-based PSCs, it is desired that the solid-state ionization potential (*I*_p_) of HTMs is around 4.9–5.5 eV [11,28–32]. The ionization energies of the investigated p-type semiconductors **BE1**–**BE7** were measured by photoemission spectroscopy in air. An example of an *I*_p_ graph is presented in electronic supplementary material, figure S7. After analysing the *I*_p_ results presented in table 2, a trend was observed: materials containing 2,2-bis(4-methoxyphenyl)ethenyl fragments tend to have lower ionization energies in comparison to their respective analogues with diphenylacetaldehyde moieties. The difference in *I*_p_ values is the result of free electron pairs of oxygen in methoxy groups that help to lower the ionization energy of the molecule [33,34]. From the energetics point of view, all of the new materials could be used in OLEDs, antimony sulfide or selenide SCs, OSCs and triple cation-based PSCs.

**Table 2.** *I*_p_ and hole mobility data for biphenyl enamines **BE1**–**BE7**, H3 and OBDA.

compound	*I*_p_, V^a^	*μ*_0_, cm^2^ V^–1^ s^–1^^b^	*μ*_h_, cm^2^ V^–1^ s^–1^^c^
H3	5.32	2.4 × 10^−3^	7 × 10^−3^
BE1	5.16	2.1 × 10^−3^	1.2 × 10^−2^
BE2	5.33	3.6 × 10^−4^	6.5 × 10^−3^
BE3	5.16	2 × 10^−4^	2 × 10^−2^
BE4	5.37	7.5 × 10^−6^^d^	5.5 × 10^−5^^d^
OBDA	5.26	8 × 10^−7^^d^	1.9 × 10^−5^^d^
BE5	5.03	1.6 × 10^−5^	4.5 × 10^−4^
BE6	5.06	2 × 10^−6^	8 × 10^−5^
BE7	5.09	5 × 10^−5^	8.8 × 10^−4^

^a^Solid-state ionization potential (*I*_p_) was measured by the photoemission in air method from films.

^b^Mobility value at zero field strength.

^c^Mobility value at 6.4 × 10^5^ V cm^–1^ field strength.

^d^Drift carrier mobility measured with PC-Z.

Another essential property for an efficient charge-transporting material is charge-carrier mobility, which determines how fast electrons or holes travel through the device. If charge mobility is low, non-radiative recombination could occur more frequently, resulting in a drop in the overall device efficiency [35]. Generally, hole-mobility values of 10^−4^ cm^2^ V^–1^ s^–1^ and higher at zero field strength are desired for OLED and SC applications [11,36], while OFET devices usually operate with mobilities of 10^−1^ cm^2^ V^–1^ s^–1^ and higher [37]. The charge-transport properties of HTMs were measured from films by the xerographic time-of-flight (XTOF) method. Charge-carrier mobility graphs of tested materials are presented in electronic supplementary material, figure S8. The values of the charge-mobility-defining parameters—zero field mobility (*μ*_0_) and the mobility at the electric field of 6.4 × 10^5^ V cm^–1^ for compounds **BE1**–**BE7**—are given in table 2. The lowest mobility is observed for **BE4**; due to the poor quality of thin film from pristine material, it had to be mixed with PC-Z at a weight ratio 1 : 1 to obtain uniform layers. Therefore, results for **BE4** are lower (by approximately one order of magnitude) due to the presence of a large portion of a nonconductive polymer. However, the mobility of **BE4** at zero electric field was still higher than that of **BE6**, which was used pristine for the mobility measurements. On the other hand, out of the new HTMs, **BE1** displayed the highest mobility at zero electric field, while results for biphenyl derivatives **BE2** and **BE3** were one order of magnitude lower.

After analysing the mobility data of the tested materials, a trend can be seen: methoxy groups tend to lower the value of drift carrier mobility. For example, **BE1** demonstrates results two orders of magnitude higher than **BE5** containing methoxy groups. The decrease in mobility is most likely caused by the less tight packing occurring due to the addition of methoxy groups, which in turn leads to longer distances between charge-hopping sites, thus slowing the charge transport.

## 3. Conclusion

In this work, seven new biphenyl enamines were synthesized using single-step reaction without the use of expensive transition metal catalysts. After measuring their thermal, optical and photophysical properties and comparing them with OSs known in literature, materials **BE1**–**BE3** emerged as the most likely candidates for applications in organic or hybrid electronics. These materials are thermally and electrochemically stable, have appropriate energy levels, and possess high drift carrier mobility, reaching 2 × 10^−2^ cm^2^ V^−1^ s^−1^ (**BE3**) at strong electric fields, making them suitable for applications in OLEDs, PSCs, OSCs and antimony selenide SCs as HTMs.

## Data Availability

All research materials supporting the data and conclusions described in the manuscript have been provided either within the main text itself or in the 16 pages of the Supporting Information section [38].
